# Professor Emma Whitelaw, a pioneer of epigenetic inheritance

**DOI:** 10.3389/freae.2025.1733711

**Published:** 2026-01-20

**Authors:** Alyson Ashe, Jessica J Hawes

**Affiliations:** School of Life and Environmental Sciences, Charles Perkins Centre, University of Sydney, Darlington, NSW, Australia

**Keywords:** ENU mouse mutagenesis, epiallele, epigenetics (chromatin remodeling), epigenetics (DNA methylation), genetic screen

## Abstract

Here we explore the contribution of Professor Emma Whitelaw to the fields of epigenetics and transgenerational epigenetic inheritance. We highlight some recent advances to the field, while integrating the work of Professor Whitelaw and her team within our current understanding of epigenetic mechanisms.

In 1942, Conrad Waddington coined the term ‘Epigenetics’ to describe the “complex of developmental processes” that bridge the genotype and phenotype (Waddington, 1942). Waddington was, first and foremost, a developmental biologist interested in the complexities of development that are recapitulated with remarkable robustness in every individual. Today, the most commonly accepted definition of epigenetics is ‘the study of changes in gene expression that do not involve alterations of the underlying DNA sequence’. However, some argue that epigenetics should only refer to cellular memory and reprogramming (Greally, 2018), while others take a much broader view of epigenetic mechanisms and their implications for a developing organism and its offspring, such as epigenetic inheritance (for conflicting opinions, see (Deichmann, 2020; Jablonka, 2017)).

While the definition of epigenetics itself is debated, so too is the precise nature of what encompasses epigenetics at a molecular level. Historically, only methylation and other post-translational modifications of DNA and the histone proteins around which it wraps were considered epigenetics (Felsenfeld, 2014). Others consider non-coding RNAs that can post- or co-transcriptionally silence genes and transcription factors to also be epigenetic factors (Mangiavacchi et al., 2023). More recently, mechanisms that regulate gene expression through the spatial and structural arrangement of chromatin in the nucleus have also been brought under the umbrella of epigenetics, such as chromatin looping, the tethering of loci together to facilitate transmission of chromatin state, and DNA supercoiling (Cavalli and Misteli, 2013; Gilbert and Marenduzzo, 2025).

Epigenetic inheritance is the idea that epigenetic signals may be passed between generations, from parent to offspring, where they can have a functional consequence in offspring. Such inheritance can be either intergenerational or transgenerational and is highly controversial. Epigenetic inheritance goes against the strict definition of Neo-Darwinism, which is the theory that evolution proceeds solely through natural selection on random mutations alone. However, attractive as the idea may be for some, there are many barriers to widespread acceptance of epigenetic inheritance. These barriers are both biological (for example, the Weismann barrier (Weismann, 1893)) and intellectual (for example, the perceived alignment between epigenetic inheritance and Lamarck’s theory of inheritance of acquired traits. For detailed reviews, see (Bird, 2024; Deichmann, 2016; Heard and Martienssen, 2014; Miska and Ferguson-Smith, 2016; Perez and Lehner, 2019)).

In this perspective, we will set aside the complexities and controversies that plague the field, and focus on one of the pioneers of mammalian epigenetic inheritance, Prof. Emma Whitelaw (who retired in the mid 2010s). Whitelaw was driven to epigenetics, like Waddington, from a developmental perspective. Specifically, she studied the regulation of globin genes in mice (Whitelaw et al., 1989). Having learnt mouse transgenesis on the weekends from her friend Rosa Beddington (a talented developmental biologist and embryologist whose life was tragically cut short in 2001 (Rastan and Robertson, 2001)), Whitelaw developed independent lines (each with a unique integration site) of transgenic mice carrying a globin gene promoter driving beta-galactosidase expression in mouse erythrocytes. She found that these lines of mice each displayed variable percentages of erythrocytes that expressed beta-galactosidase, but that this variegation was highly consistent within a line (Robertson et al., 1995), a phenomenon strikingly similar to position-effect variegation (PEV) in the eyes of *Drosophila* (Reuter and Spierer, 1992). Furthermore, she showed that this stochastic expression appeared to be driven by the integration site and by chromatin accessibility, but not DNA methylation (Garrick et al., 1996).

Intrigued by the stochastic nature of the silencing she observed in the erythrocytes, she began working on the agouti viable yellow (A^vy^) strain of mice. These mice have coats that vary between individuals from completely yellow to completely agouti, with a full spectrum of variegation in between. This mouse line was molecularly characterized by others (Duhl et al., 1994; Wolff et al., 1998) but Whitelaw and her team noticed that the spectrum of yellow colouration in the coats of offspring was, in part, heritable and dependent on the phenotype of the mother and grandmother (Morgan et al., 1999). A series of elegant experiments confirmed that this effect was not due to the uterine environment or an effect on the oocytes before fertilisation, suggesting that an epigenetic signal was being inherited from one generation to the next (and the next). Furthermore, they showed that the degree of yellow in the coat correlated with DNA methylation at an intracisternal-A particle (IAP) retrovirus inserted just upstream of the agouti gene. Subsequent experiments suggested that this DNA methylation could not be the sole cause of the epigenetic inheritance, as blastocysts from both yellow and pseudoagouti mothers displayed complete erasure of DNA methylation at the agouti locus (Blewitt et al., 2006). The A^vy^ mice were the first example of epigenetic inheritance in mammals and have served as the poster child for the field ever since, with pictures of mice with differing coat colours being incorporated in many undergraduate lectures and conference presentations on epigenetics.

Shortly after this paper, Whitelaw and her team published two more papers identifying more instances of epigenetic inheritance and parental effects in mice (at the *Axin* ^*Fu*^ allele, and once again in erythrocytes, this time with a GFP reporter (Preis et al., 2003; Rakyan et al., 2003)). Similarly to the *A* ^*vy*^ allele, they showed that the *Axin* ^*Fu*^ allele was associated with variable methylation at an IAP (intracisternal A-particle) element, in this case inserted into intron 6 of the *axin* gene (Rakyan et al., 2003). They coined the term metastable epialleles to describe such loci of variable penetrance (Rakyan et al., 2002) in which the epigenetic landscape at a given sequence varies between individuals, but is consistent across the tissues of an individual, somewhat like an epigenetic fingerprint. It is worth nothing that subsequent work by the lab of Anne Ferguson-Smith has shown that *A* ^*vy*^ and *Axin* ^*Fu*^ are not the norm: although a subset of IAP elements display variable methylation patterns, and an even smaller subset display low penetrance of epigenetic inheritance, most display robust reprogramming of their methylation between generations (Elmer et al., 2021; Kazachenka et al., 2018). Similar results were also found recently by the Waterland lab in a genome-wide screen for metastable epialleles (Gunasekara et al., 2025).

Driven by a desire to understand more about the underlying biology of these puzzling phenomena and inspired by similar screens for modifiers of PEV in *Drosophila* and paramutation in maize that showed genetic control of epigenetic phenomena (Hollick and Chandler, 2001; Schotta et al., 2003), Whitelaw and her team embarked on a set of ambitious ENU mutagenesis screens (the Momme screens, meaning *Modifiers of murine metastable epialleles*) (Ashe et al., 2008; Blewitt et al., 2005; Daxinger et al., 2012; Daxinger et al., 2013). The first Momme screen was performed when large-scale ENU mutagenesis screens had only recently been developed in mice (de Angelis et al., 2000; Nolan et al., 2000; Soewarto et al., 2000) and took advantage of a variegating erythrocyte GFP reporter mouse line (Preis et al., 2003). Crucially, this enabled relatively fast and simple FACS-based screening of potential mutants. The goal of these ENU screens was to discover the genes controlling epigenetic regulation of gene expression, with the hypothesis that many of these genes would have fundamentally important roles in epigenetic control of development, and potentially epigenetic inheritance. And indeed, this is what they found.

Although the ENU mutagenesis screens were performed on a transgene expressed in erythrocytes, the mutations discovered spanned the breadth of what are now characterised as epigenetic processes (Figure 1; Table 1). It is unsurprising, given what we currently know about gene silencing, that DNA methyltransferases were amongst the Momme mutants (Ashe et al., 2008; Whitelaw et al., 2010; Youngson et al., 2013). Indeed, *MommeD2*, the second Momme line to be isolated, was shown in 2007 to contain a probable loss-of-function allele of *Dnmt1* (Chong et al., 2007). Although the methylation status at the GFP transgene was not characterised, *Dnmt1* ^*MommeD2/+*^ mice showed an increased proportion of yellow offspring when crossed to the *A* ^*vy*^ mouse line, consistent with a loss of silencing resulting from less DNA methylation at the locus (Blewitt et al., 2005; Chong et al., 2007).

**FIGURE 1:**
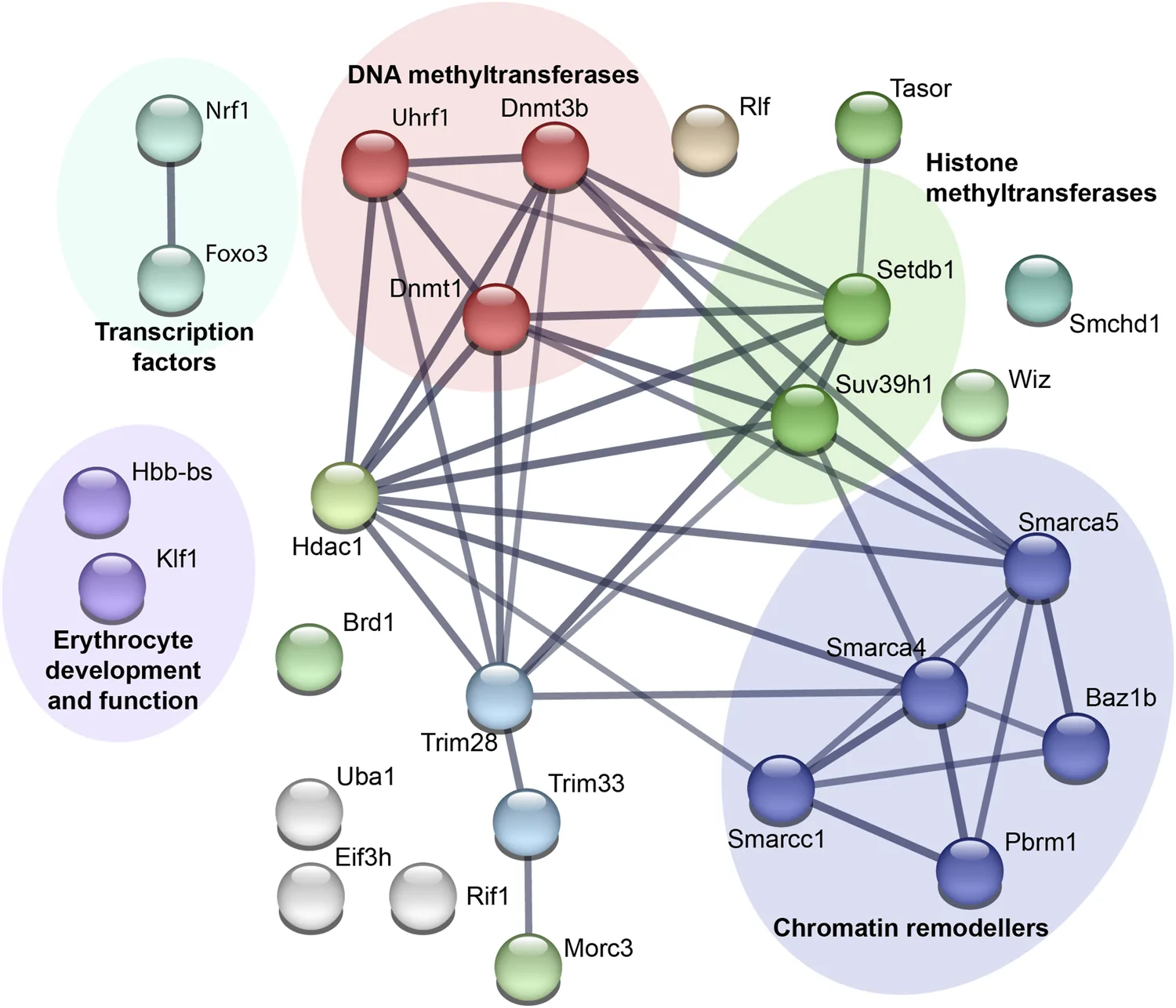
A string network of the identified hits from the Momme screens. Nodes represent proteins and are clustered and colored based on known function or activity. The darkness of the lines between nodes indicates the confidence of the associations (darker line indicates higher confidence). Associations do not necessarily indicate physical binding, but rather a shared or similar function. Generated using the STRING database (Szklarczyk et al., 2023).

**TABLE 1:** A summary of the genes found in the ENU mutagenesis screens performed by the Whitelaw Lab. *D* refers to genes found in dominant screens and *R* to those found in recessive screens. The table includes only those lines that have currently been mapped, and not those that remain unknown.

Momme mutant	Gene	Protein function	References for momme mutant(s)
*D10, D16*	*Baz1b*	Tyrosine-protein kinase BAZ1B. Component of chromatin remodelling complex (Oppikofer et al., 2017)	Ashe et al. (2008), Daxinger et al. (2013)
*D42*	*Brd1*	Bromodomain-containing protein 1. Interacts with histone acetyltransferases and methyltransferases (Mishima et al., 2011)	Daxinger et al. (2013)
*D2, D32*	*Dnmt1*	DNA methyltransferase 1. Maintains CpG methylation over DNA replication	Chong et al. (2007), Daxinger et al. (2013)
*D14*	*Dnmt3b*	DNA methyltransferase 3b. Involved in establishing CpG methylation *de novo*	Youngson et al. (2013)
*D12, D38*	*eIF3h*	Component of EIF3 complex. Required for the initiation of protein synthesis (Masutani et al., 2007)	Daxinger et al. (2012)
*D6, D20*	*Fam208a*	TASOR. Component of HUSH complex, involved in silencing retrotransposons (Douse et al., 2020)	Harten et al. (2014)
*D7*	*Hbb-b1*	Hemoglobin subunit beta-1. Involved in oxygen transport	Brown et al. (2013)
*D5*	*Hdac1*	Histone deacetylase 1. Component of multiple chromatin modifying and remodelling complexes, such as NuRD (nucleosome remodelling complex)	Ashe et al. (2008), Blewitt et al. (2005)
*D11, D45*	*Klf1*	Krueppel-like factor 1. Transcriptional regulator for beta-globin gene promoter	Sorolla et al. (2015)
*D41*	*Morc3*	MORC family CW-type zinc finger protein 3, binds H3K4me0/1/2/3 and has ATPase activity which facilitates phase separation (Zhang et al., 2019)	Desai et al. (2021)
*D46*	*Nrf1*	Nuclear respiratory factor 1. CNC-bZIP transcription factor essential for development and maintenance of homeostasis, involved in DNA hypomethylation	Sorolla et al. (2021)
*D27*	*Pbrm1*	Protein polybromo-1. Component of PBAF SWI/SNF chromatin remodelling complex (Hao et al., 2025)	Daxinger et al. (2013)
*D18*	*Rif1*	Rap-interacting factor 1. Involved in DNA damage response to promote repair of double-strand breaks (Kumar and Cheok, 2014)	Daxinger et al. (2013)
*D8, D26, D28, D34*	*Rlf*	Rearranged L-myc fusion. Necessary for DNA hypomethylation at specific loci, likely involved in development through NOTCH signalling (Bourke et al., 2017)	Daxinger et al. (2013)
*D13, D17*	*Setdb1*	Histone-lysine N-methyltransferase SETDB1. Methylates H3K9, especially at repetitive elements and developmental genes (Padeken et al., 2022)	Daxinger et al. (2013)
*D39*	*Smarca4*	SWI/SNF related, matrix associated, actin dependent regulator of chromatin, subfamily a, member 4. ATPase subunit of SWI/SNF chromatin remodelling complex (Ahmad et al., 2024)	Daxinger et al. (2013)
*D4, D35, D37*	*Smarca5*	SWI/SNF-related matrix-associated actin-dependent regulator of chromatin subfamily A member 5. ATPase component of imitation switch (ISWI) chromatin remodelling complex (Goodwin and Picketts, 2018)	Blewitt et al. (2005), Chong et al. (2007), Daxinger et al. (2013)
*D19*	*Smarcc1*	SWI/SNF related, matrix associated, actin dependent regulator of chromatin, subfamily c, member 1. Component of BAF complex from SWI/SNF family of chromatin remodellers (Ahmad et al., 2024)	Daxinger et al. (2013)
*D1, D23, D36, D43*	*Smchd1*	Structural maintenance of chromosomes flexible hinge domain-containing protein 1. Epigenetic repressor involved in X-chromosome inactivation (Huang et al., 2025)	Blewitt et al. (2005), Blewitt et al. (2008), Daxinger et al. (2013), Tapia del Fierro et al. (2023)
*D33*	*Suv39h1*	Histone-lysine N-methyltransferase Suppressor of variegation 3-9 homolog 1. Histone methyltransferase involved in H3K9 methylation to establish pericentric heterochromatin and *de novo* gene silencing (Padeken et al., 2022)	Daxinger et al. (2013)
*D9, D31*	*Trim28*	Tripartite motif protein 28. E3 ubiquitin protein ligase, involved in formation of chromosome loops, can recruit chromatin remodelling complexes and histone modifiers (Czerwińska et al., 2017; Wei et al., 2025)	Daxinger et al. (2013), N. C. Whitelaw et al. (2010)
*D44*	*Trim33*	Tripartite motif protein 33/TIF1γ. E3 ubiquitin protein ligase, can promote transcriptional elongation and DNA damage repair (He et al., 2006; Yu et al., 2019)	Isbel et al. (2015)
*D40*	*Uhrf1*	Ubiquitin-like with PHD and RING finger domains 1. Maintains DNA methylation through interactions with DNMT1, can bind methylated DNA and histone H3K9me2/3 (Ferry et al., 2017)	Daxinger et al. (2013)
*D30*	*Wiz*	Widely interspaced zinc finger-containing protein. Involved in heterochromatin formation through recruitment of histone methyltransferases, associates with DNA loop anchors alongside CTCF and Cohesin (Justice et al., 2020)	Daxinger et al. (2013), Isbel et al. (2016)
*R1*	*Foxo3a*	Forkhead box protein O3. ‘Winged helix’ or ‘Forkhead’ transcription factor, regulates development and cell-cycle control (Cao et al., 2023)	Youngson et al. (2011)
*R2*	*Uba2*	SUMO-activating enzyme subunit 2. Mediates ATP-dependent activation of SUMO proteins necessary for genome stability, nuclear organisation and maintaining repressive chromatin states (Poleshko et al., 2014)	Ashe (2007)

Other mutants were more enigmatic at the time of discovery, and some remain mysterious. For example, numerous chromatin writers, readers and remodellers were hit (Ashe et al., 2008; Blewitt et al., 2005) when the ‘histone code’ theory had only recently been proposed (Strahl and Allis, 2000). For example, *MommeD10* mutant mice were shown to have a mutation in *Williams syndrome transcription factor/Baz1b*. DNA methylation at the transgene locus was not altered in *MommD10* ^*+/−*^ or *MommeD10* ^*−/−*^ mice, and so the hypothesis was made that changes to transgene expression were caused by alterations at the chromatin level (Ashe et al., 2008). BAZ1B encodes a protein containing a bromodomain and PHD finger, two domains commonly associated with chromatin binding, and is a subunit of the SMARCA5 chromatin remodelling complex (Oppikofer et al., 2017). It also plays an important role in DNA repair (Aydin et al., 2014; Kim et al., 2024), and heterochromatin replication (Bozhenok, 2002; Goto et al., 2024), although at the time of its discovery as *MommeD10*, most of this was not known. Importantly, *MommeD10* ^*−/−*^ mice showed craniofacial abnormalities reminiscent of humans with Williams-Beuren syndrome, and this was consistent with its expression pattern in mouse embryos, suggestive of a causative role for BAZ1B in this syndrome (Ashe et al., 2008).

Another of the first Mommes, *MommeD1*, was discovered to have a mutation in a previously uncharacterised gene, Structural maintenance of chromosomes hinge domain 1 (*Smchd1)* and was shown to be critical for X-inactivation (Blewitt et al., 2008). This discovery led to a plethora of research defining the role of *Smchd1* not only in X inactivation, but also facioscapulohumeral muscular dystrophy and bosma arrhinia microphthalmia syndrome (reviewed in (Gurzau et al., 2020; Schall et al., 2019)) and most recently in heterochromatin maintenance more broadly (Huang et al., 2025). Yet another gene discovered in the Momme screen (*MommeD8, D28 and D34*), and not studied previously, *rearranged L-Myc fusion (Rlf)*, helps to regulate DNA methylation levels at CpG islands (Harten et al., 2015), and its loss leads to developmental defects in the heart (Bourke et al., 2017). These *Momme* mutants (*D1, D10, D8*) collectively serve as examples of how the mutagenesis screen led directly to subsequent advances in our understanding of the role of epigenetic modifiers in development, in ways that could not have been predicted at the outset of the screen.

Many of the hits are involved not only in epigenetic regulation during development, but also current and emerging mechanisms of epigenetic inheritance. For example, TASOR (Fam208a), identified from the Momme screen more recently (Harten et al., 2014), is part of the HUSH complex which recruits SETDB1 (another Momme mutant (Daxinger et al., 2013)), to deposit H3K9me3 (Douse et al., 2020). Though H3K9me3 profiles were not studied in the initial Momme screens, we now know that DNA methylation and H3K9me3 work closely together and are both needed for effective and heritable silencing (Tatarakis et al., 2025). MORC3 is another Momme mutant (Desai et al., 2021) and has been identified as required for the recruitment of histone H3.3, a variant of H3, to maintain H3K9me3 and heterochromatic silencing at endogenous retroviruses (ERVs) (Groh et al., 2021). Very recently, the overexpression of H3.3 and its transport from somatic cells to the germline was observed in *C. elegans* as both a stimulus and potentiator of the transgenerational epigenetic inheritance of lysosomal lipolysis phenotypes (Zhang et al., 2025). These data highlight the regulation and trafficking of histone genes as emerging mechanisms of epigenetic regulation and inheritance.

TRIM28 is another interesting candidate from the screen, characterised as a bridge between histone deacetylases (via the NuRD complex), transcription factors, and histone methyltransferases (Czerwińska et al., 2017). Haploinsufficiency of *Trim28* can trigger a bivalent epigenetic switch leading to obesity in a subset of mice within an isogenic population (Dalgaard et al., 2016; Whitelaw et al., 2010), and very recently, TRIM28 was linked to 3D genome organisation at chromosomal loops, necessary for the activation of CD8^+^ T-cells (Wei et al., 2025). TRIM33 is another tripartite motif containing protein identified in the Momme screen. It targets one of the youngest mouse retrotransposons in the germline, RLTR10B-containing LTRs, surveilling their activity (Isbel et al., 2015). TRIM28 and TRIM33 interact with TRIM27, a transcriptional regulator involved in early genome activation (Torres-Padilla and Zernicka-Goetz, 2006), to form a complex that can modulate hepatocellular carcinoma, one of the most common types of cancers worldwide (Herquel et al., 2011).

In the 15 years since Prof. Whitelaw and Dr Lucia Daxinger asserted that the study of transgenerational epigenetic inheritance had raised ‘more questions than answers’ (Daxinger and Whitelaw, 2010), have we made headway in finding solutions? The study of epigenetics has benefited from advancements in sequencing technologies, enabling lower input and higher accuracy in chromatin profiling, motif discovery, the introduction of spatial methods, and an ever-increasing library of protein and nucleic acid post-translational modifications (discussed in (Henikoff, 2023)). This has also facilitated precision epigenetic editing, such as through dCas9-fusions and TALEs (reviewed in (Roth et al., 2024)). Experiments using these editors and other thoughtfully engineered molecular tools have probed the heritability of epigenetic mechanisms *in vivo*, showing that marks such as DNA methylation, small RNAs and specific histone modifications can indeed be maintained across generations (Argaw-Denboba et al., 2024; Herridge et al., 2025; Takahashi et al., 2023).

It is widely accepted that transgenerational epigenetic inheritance can occur in invertebrates, such as *C. elegans* and *D. melanogaster* (reviewed in (Santilli and Boskovic, 2023)) and in plants (reviewed in (Cao and Chen, 2024)). The most prominent controversy still surrounds mammals (Bird, 2024). The epigenome appears, by nature, to be sensitive to subtle environmental perturbations, from social stress (Gapp et al., 2014; Weaver et al., 2004) to temperature (reviewed in (Murray et al., 2022)). Following molecular changes across generations is complicated by numerous confounders, including many we have yet to identify, making tracking inheritance difficult even in mouse models (Heard and Martienssen, 2014; Sapozhnikov and Szyf, 2024). In addition, negative results can be hard to publish, as pointed out by Whitelaw herself in 2015 (Whitelaw, 2015). Human studies are virtually impossible to perform in a properly controlled manner (Horsthemke, 2018), and studying transgenerational epigenetic inheritance requires multiple lifespans. Nonetheless, epidemiological studies on humans are suggestive of epigenetic inheritance (Kaati et al., 2007; Pembrey et al., 2006) and provide a context in which epigenetic inheritance could fit into adaptation, if one takes a broad view of epigenetic mechanisms (Jablonka, 2013).

Ultimately, as evidenced by the pioneering work of Prof. Emma Whitelaw and many of her peers, epigenetic mechanisms are multifaceted and complex. As we develop more sophisticated experimental tools, we are starting to see how some of the mutations from Whitelaw and colleagues’ mutagenesis screens could integrate into an architecture of epigenetic inheritance. Nonetheless, we believe epigenetics will remain an area where the questions consistently outnumber answers. Perhaps, as Whitelaw herself provocatively says, we even need new terminology: once we understand the molecular details of a phenomena, should it be classified as epigenetic anymore? (E. Whitelaw, pers. comm.).

## Data Availability

The original contributions presented in the study are included in the article/supplementary material, further inquiries can be directed to the corresponding author.
